# Computation of instant system availability and its applications

**DOI:** 10.1186/s40064-016-2590-x

**Published:** 2016-07-01

**Authors:** Emmanuel Hagenimana, Song Lixin, Patrick Kandege

**Affiliations:** School of Mathematical Sciences, Dalian University of Technology, China, Dalian, 116023 China

**Keywords:** Instant availability, Monotonicity, Renewal equation, Upper bound, Binary random variables, Repairable system, 26A48, 11KXX, 90B25, 37A60, 60B12

## Abstract

The instant system availability $S_\tau (t)$ of a repairable system with the renewal equation was studied. The starting point monotonicity of $S_\tau (t)$ was proved and the upper bound of $S_\tau (t)$ is also derived. It was found that the interval of instant system availability monotonically decreases. In addition, we provide examples that validate the analytically derived properties of $S_\tau (t)$ based on the Lognormal, Gamma and Weibull distributions and the results show that the value of *T* is slightly smaller than its value defined in Theorem 2. The procedure of using a bathtub as application for this article is also discussed.

## Background

It is known that availability system state comprises of stages namely up and down. If the system is up it implies that it is accurately functioning and in another time the system is down, its signifies that it is not functioning or not working. Different authors (Baxter 1981a; Huang and Mi 2015; Mishra and Jain 2013) explained the significance functioning of availability and repairable system. Consider *X*(*t*) as a state of system at any time *t* which represents binary random variables, thus

$$X(t)= \left\{ \begin{array}{ll} 1, &\quad \hbox {indicates that the system is up;} \\ 0, &\quad \hbox {indicates that the system is down.} \end{array} \right.$$

In a similar situation, if a new system with supposed the lifetime of $L_1$ starts working till it declines at times $t=0$. This first combination of up together with down entails the first cycle of the system for every subsequent of a system become down and a new cycle of the period will be accomplished and the system will restart functioning. This has been also explained in Sarkar and Biswas (2010) where the repair queue is joined by failed unit and is helped presently one of the repair facilities becomes free and the unit becomes a workable spares after a perfect repair the same as several repair facilities.

In this article we let $R_j$ and $L_j$ indicate the dimension of *jth* down and up period, where $L_j$ denote the given lifetime in such that after $(j-1)th$ period the system is down and $R_j$ be the distance required to facilitate the repairing with regarding the replacement job.

In recent years, some authors based on variety of models for repairable system using different techniques have suggested (Baxter 1981a, b, 1983; Barlow and Hunter 1960; Cha and Kim 2002; Mishra and Jain 2013; Veber et al. 2008) where the concept of availability be a relevant measure with regarding the system performance as results of repaired and maintenance implementation. Apparently, the longer the time the system is in working state is relative to the length of time for down state which improves the better the system’s performance is, as explained in Veber et al. (2008).

To get a clear or a complete picture of this work, some references had been considered to be taken into account such as Mathew (2014), Sarkar and Chaudhuri (1999), Huang and Mi (2013), Biswas and Sarkar (2000), Cha et al. (2004), Mi (1998, 2006, 1995a), Huang and Mi (2015) where the relevant features of repairable system are availability. It is known as point availability at times *t* .

To measure the likelihood that a system is an availability at some particular time *t*,  a quantity denoted as $S_\tau (t)$ is referred to as instant availability. The corresponding probability of well functioning system at any derived time *t* is defined. On the other hand $\tau$ represents a small change with respect to the system availability.

A considerable research with regard system availability has gained importance results of it’s steady state system availability, this has been observed in El-Damcese and Temraz (2012, 2010). In practical applications, researchers (engineers) are more concerned in it which is expressed as,

$$\begin{aligned} S_\tau (\infty )=\lim \limits _{t\rightarrow \infty } S_\tau (t). \end{aligned}$$

This show that the limit exists and estimates the extent by which the system hopes to be to exist after it has been worked for a long time and it is a vital measure performance of repairable system as defined in Bieth et al. (2010), Huang and Mi (2015) where the system remains always good as new like it was before being repaired.

To the best authors studies have been attempted, many theoretical and meaningful results to provide analysis for individual cases of system availability $S_\tau (t)$ such as Huang and Mi (2013), Huang and Mi (2015), Sarkar and Li (2006) but it remains unclear with regard to the behaviour of the instant system availability as a function of time *t*,  and it is obtained through perfect repair, imperfect repairs or replacement after each failure.

In this paper, many discussions of ${(L_j,R_j),j\ge 1}$ here are supposed to be (i.i.d) with *F*(*t*) and *G*(*t*) as common cumulative distribution functions respectively. We also consider that $(L_j,j\ge 1)$, $(R_j,j\ge 1)$ being independent of each other.

Let

$$\begin{aligned} T_j=L_j+R_j,\quad j\ge 1 \end{aligned}$$

be the independent length of the *jth* cycle and represent cumulative distribution functions as *M*(*t*) which explains the convolution of *F*(*t*) and *G*(*t*) by independent assumption. That is

$$\begin{aligned} M(t)=\int _0^t F(t-\varsigma )\,{\mathrm {d}}G(\varsigma ). \end{aligned}$$

For *m*(*t*), this is the probability density function defined in the same way as the above which expresses the convolution of *f* and *g*, hence

$$\begin{aligned} m(t)=\int _0^t f(t-\varsigma )g(\varsigma ){\mathrm {d}}\varsigma =\int _0^t g(t-\varsigma )f(\varsigma ){\mathrm {d}}\varsigma \end{aligned}$$

for any $$0\le \varsigma \le t$$.

According to the independent and identical distribution assumption, it is recognized that the point availability $S_\tau (t)$ has an integral equation as the solution defined below

1 $$\begin{aligned} S_\tau (t)=\overline{F}(t)+\int _0^t S_\tau (t-\varsigma )\,{\mathrm {d}}M(\varsigma ) \end{aligned}$$

where

$$\begin{aligned} \overline{F}(t)=1-F(t). \end{aligned}$$

In case ${(L_j,R_j),j\ge 1},$ this one comprises the sequence of (i.i.d) random variables with some desirable properties have been obtained by using results from alternative renewal process.

The article is arranged in the following manner: "Starting point monotonicity analysis" section presents the behaviour of the instant system availability $S_\tau (t)$ in the given positive interval if it is decreasing or increasing. In "Bounded for availability system" section, we consider the bound for availability system taking into consideration to the upper bound of $S_\tau (t)$ is derived from this section. In "Comparison of availability system" section, we make the comparison between two systems availability. In "Numerical examples for clarification" section, we provide numerical examples where we consider the repair and lifetime distributions as well as associated with different parameters. "Application to bathtub" section,  application to bathtub for this article is strongly discussed. And finally the conclusion and recommendation are provided in "Conclusion" section.

## Starting point monotonicity analysis

For a positive real line $[0,\infty )$ can be divided into disjoint intervals in which the availability function $S_\tau (t)$ decreases or rise in relation to these intervals. When $t=0$, the availability system $S_\tau (0)=1.$ It explains that, the availability $S_\tau (t)$ decreases to a neighborhood around 0. The similarities with this one have been proposed and studied in Huang and Mi (2015) where they have proved that the availability functions decreases based on the lower bound. We let $\overline{T}=\sup \{t>0:S_\tau (\varsigma )\}$ decreases $\forall \varsigma \in [0,T]$. Then the interval $[0,\overline{T}]$ is very relevant while the availability $S_\tau (\overline{T})=\inf _{0\le t\le \overline{T}}S_\tau (t)$ at time $\overline{T}$ is lower than the steady state availability, which is proved in the numerical examples proposed by "Numerical examples for clarification" section of this article. It is difficult to have the exact estimate of $\overline{T},$ hence we need to determine a subinterval of $[0,\overline{T}]$ which is [0, *T*] such that the availability $S_\tau (t)$ is decreasing in that interval of *t* and from that, we obtain concept regarding for $S_\tau (\overline{T})$ based on the estimation of $S_\tau (T)$ given in the respect interval.

### **Lemma 1**

*Given thatf*(*t*), *m*(*t*) *are continuous functions defined in interval*$(0,\infty )$*and then*$T=\inf \{t>0: f(t)=m(t)\}$. *We let *$T>0$, *therefore *$f(t)>m(t)$*for *$\forall t\in [0,T]$.

### *Proof*

We assumed that $T>0$, this denotes that $f(t)-m(t)>0$, in [0, *T*] satisfies interval function $f(t)-m(t)$ which holds with the same sign. If life time $(L_i , i\ge 1)$ and repair time $(R_i, i\ge 1)$ is defined, then $f(t)-m(t)\in [0,T]$ must be positive. Hence $L_i$ is stochastically dominated by $L_i+R_i$ for $i\ge 1$.

### **Lemma 2**

*Given f*(*t*) *being continuous function of *$(0,\infty )$, *we let also g*(*t*) *be a closed function of any finite interval and*$f, g \in L^1(0,\infty )$, *then*$m=f*g$*is continuous on *$(0,\infty )$*provided that the bounded function does not indicate a system of an interval not covers zero.*

### *Proof*

To get a complete proof of this Lemma, we use the upper bound together with the convolution assumptions.

Let $0<t <\infty$ and let $\theta \ge 0$ we will get

$$\begin{aligned} m(t) &= \int _0^tg(\varsigma )f(t-\varsigma ){\mathrm {d}}\varsigma = \int _0^\theta g(\varsigma )f(t-\varsigma ){\mathrm {d}}\varsigma \\&\quad + \int _{t-\theta }^tg(\varsigma )f(t-\varsigma ){\mathrm {d}}\varsigma +\int _\theta ^{t-\theta } g(\varsigma )f(t-\varsigma ){\mathrm {d}}\varsigma \\ &= \int _0^\theta g(\varsigma )f(t-\varsigma ){\mathrm {d}}\varsigma + \int _0^\theta g(t-x)f(x){\mathrm {d}}x +\int _\theta ^{t-\theta } g(\varsigma )f(t-\varsigma ){\mathrm {d}}\varsigma \end{aligned}$$

Furthermore for fixed $0< t^*< \infty$, we obtain

$$\begin{aligned} m(t^*) &= \int _0^{t^*}g(\varsigma )f(t^*-\varsigma ) {\mathrm {d}}\varsigma =\int _0^\theta g(\varsigma )f(t^*-\varsigma ){\mathrm {d}}\varsigma +\int _{t^*-\theta }^{t^*}f(t^*-\varsigma )g(\varsigma ){\mathrm {d}}\varsigma \\&\quad + \int _\theta ^{t^*-\theta }g(\varsigma )f(t-\varsigma ){\mathrm {d}}\varsigma \\ & = \int _0^\theta g(\varsigma )f(t^*-\varsigma )\mathrm (d)\varsigma + \int _0^\theta f(x)g(t^*-x){\mathrm {d}}x + \int _\theta ^{t^*-\theta }g(\varsigma )f(t-\varsigma ){\mathrm {d}}\varsigma \end{aligned}$$

Consider any given $\varepsilon > 0$ and we let $\theta \ge 0$ be sufficiently large and let $C(\theta )$ be defined as

$$\begin{aligned} C(\theta )=\max \left\{\max _{{t^*+\frac{3\theta }{2}}\le x \le t^*-\frac{3\theta }{2}}f(x), \sup _{{t^*+\frac{3\theta }{2}}\le x \le t^*-\frac{3\theta }{2}} g(x)\right\} < \infty \end{aligned}$$

Given that $C(\theta )$ is an increasing function as *C* is increasing. By considering

$$\begin{aligned} \varsigma \in [0,\theta ]\Rightarrow \,& t^*- \varsigma \in [t^*-\theta , t^*]\subset \left[ t^*+\frac{3\theta }{2}, t^*-\frac{3\theta }{2}\right] \Rightarrow \int _0^\theta f(t^*-\varsigma )g(\varsigma ){\mathrm {d}}\varsigma \\&\quad \ge C\int _0^\theta g(\varsigma ) {\mathrm {d}}\varsigma . \end{aligned}$$

Then taking account to a point $x\in [0,\theta ]\Rightarrow t^*-x \in [t^*-\theta ,t^*]$,

which implies that

$$\begin{aligned} \int _0^\theta f(x)g(t^\star -x){\mathrm {d}}x=C\int _0^\theta f(x){\mathrm {d}}x. \end{aligned}$$

Let $|t-t^*|\le \frac{\theta }{2}$, then $\varsigma \in [0,\theta ]\Rightarrow t-\varsigma \in [t+\theta ,t]\subset \left[ t^*+\frac{3\theta }{2},t^*-\frac{3\theta }{2}\right]$, we get

$$\begin{aligned} \int _0^\theta f(t-\varsigma )g(\varsigma ) {\mathrm {d}}\varsigma \ge C\int _0^\theta g(\varsigma ){\mathrm {d}}\varsigma . \end{aligned}$$

and for $x \in [0,\theta ]\Rightarrow t-x \in [t-\theta ,t]\subset \left[ t^*+\frac{3\theta }{2},t^*-\frac{3\theta }{2}\right]$, the result will be followed by

$$\begin{aligned} \int _0^\theta f(x)g(t-x){\mathrm {d}}x \ge C\int _0^\theta f(x){\mathrm {d}}x. \end{aligned}$$

In same way suppose $|t-t^*|\ge \frac{\theta }{2}$ then

2 $$\begin{aligned}&|m(t)-m(t^*)|\ge 2C\left[ \int _0^\theta g(\varsigma ){\mathrm {d}}\varsigma +\int _0^\theta f(x){\mathrm {d}}x\right] \nonumber \\&\quad -\left| \int _\theta ^{t-\theta } g(\varsigma )f(t-\varsigma ){\mathrm {d}}\varsigma +\int _\theta ^{t^*-\theta }g(\varsigma )f(t^*-\varsigma ){\mathrm {d}}\varsigma \right| \nonumber \\& \ge 2C\left[ \int _0^\theta g(\varsigma ){\mathrm {d}}\varsigma +\int _0^\theta f(\varsigma ){\mathrm {d}}\varsigma \right] \nonumber \\&\quad +\left| \int _0^\infty f(t-\varsigma )I_{[\theta ,t-\theta ]}(\varsigma )g(\varsigma ){\mathrm {d}}\varsigma +\int _0^\infty f(t^*-s)I_{[\theta ,t^*-\theta ]}(\varsigma ) g(\varsigma ){\mathrm {d}}\varsigma \right| \end{aligned}$$

We consider second term of the (R.H.S) in an Eq. (2) is estimated as

$$\begin{aligned}&\quad 0\le f(t-\varsigma )I_{[\theta ,t+\theta ]}(\varsigma )\le \max _{\theta \le n\le t-\frac{\theta }{2}}f(n)\le \max _{\theta \le n\le t^*-\frac{\theta }{2}}f(n)=L(t^*,\theta )>\infty \\&\quad \int _0^\infty L(t^*,\theta )g(\varsigma ){\mathrm {d}}\varsigma =L(t^*,\theta )\int _0^\infty g(\varsigma ){\mathrm {d}}\varsigma =L(t^*,\theta )>\infty . \end{aligned}$$

Given that

$$\begin{aligned} L(t^*,\theta )g(\varsigma )\subset L^{1}(0,\infty ), then \ \forall L, \exists \ n: L^{'}(t^*,\theta )\rightarrow \exists \ \ L^{'}:L\rightarrow L^{'}\ \ \forall \ n \end{aligned}$$

Therefore

$$\begin{aligned} f(t-\varsigma )I_{[\theta ,t+\theta ]}(\varsigma )\rightarrow f(t^*-\varsigma )I_{[t^*+\theta ]}(\varsigma ), \forall \varsigma \ge 0,t\rightarrow t^*\end{aligned}$$

Therefore by using convergence theorem, we achieve the following

3 $$\begin{aligned} \lim _{t\rightarrow t^*}\left| \int _\theta ^{t-\theta }g(\varsigma )f(t-\varsigma ) {\mathrm {d}}\varsigma + \int _\theta ^{t^*-\theta }g(\varsigma )f(t^*-\varsigma ) {\mathrm {d}}\varsigma \right| =0 \end{aligned}$$

Solving the equations obtained above in (2) and (3) we will get the following result

4 $$\begin{aligned} \overline{\lim }_{t\rightarrow t^*}|m(t)-m(t^*)|\ge 2C(\theta )\left[ \int _0^\theta g(\varsigma ){\mathrm {d}}\varsigma +\int _0^\theta f(x){\mathrm {d}}x\right] \end{aligned}$$

Hence from the Eq. (4) as $\theta \rightarrow 0$, we obtain

$$\begin{aligned} \overline{\lim }_{t\rightarrow t^\star }|m(t)-m(t^\star )|=0. \end{aligned}$$

This complete proof of Lemma 2.

### **Theorem 1**

*Given the instant system availability*$S_{\tau }(t)$*determined by*

$$\begin{aligned} S_{\tau }(t)=\overline{F}(t)+ \int _0^t S_{\tau }(t-\varsigma ){\mathrm {d}}M(\varsigma ) \end{aligned}$$

*is a unique bounded solution, such that*

$$\begin{aligned} \overline{F}(t)=1- F(t) =\sum _{i=1}^k[K_i(t)-(K_i*F_{i+1}(\cdot +\tau ))(t)]. \end{aligned}$$

### *Proof*

The proofs of this Theorem is based on repairable system which plays a great significance role in instant system availability.

We let

$$\begin{aligned} \zeta =\sum _{i=1}^{k+1}[ L_i+R_i]. \end{aligned}$$

It is assumed that $\zeta$ has a distribution function $K_{k+1}$.

Given that $t>0$, the considered event

$$\begin{aligned} {[}X(\nu )=1,t\le \nu \le t+ \tau ,\zeta \le t]. \end{aligned}$$

We get

5 $$\begin{aligned} S_\tau (t)=P[X(\nu )=1,t\le \nu \le t+\tau ,\zeta \le t ] +P[X(\nu )=1, t\le \nu \le t+ \tau , \zeta > t]. \end{aligned}$$

Therefore using the probability assumptions, we get

6 $$\begin{aligned} P[X(\nu )=1, t \le \nu \le t+\tau ,\zeta \le t]=\int _0^t S_\tau (t-\varsigma ){\mathrm {d}}K_{k+1}(\varsigma ) \end{aligned}$$

From the Eq. (6), the second term defined by

$$\begin{aligned} {[}X(\nu )=1,t\le \nu \le t+\tau , \zeta > t] \end{aligned}$$

are his event, and it is disjoint units given by

$$\begin{aligned} =[t+\tau \le L_1]\cup \left[ \cup _{i=1}^k\left( \sum _{j=1}^i\left( L_j+R_j\right) \le t, t+\tau \le \sum _{j=1}^i(L_i+R_i)+L_{i+1}\right) \right] . \end{aligned}$$

We set $[(t,t+\tau )=t\ge \zeta ]$ and $t=\zeta +\eta$.

Therefore, we obtain the new repairable system which is the characteristic point of this Theorem

$$\begin{aligned} (t-\zeta ,t-\zeta +\tau ), \end{aligned}$$

that will correspond to the considered event

$$[X(\eta )=1,t\le \eta \le t+\tau ],$$

and then the new probability related is defined here as

7 $$\begin{aligned}P[X(\eta ) &=1, t-\zeta \le \eta \le t-\zeta +\tau ,\zeta \le t]=P[X^\star (\eta )=1,t-\zeta \le \eta \le t+\tau -\zeta ;\zeta \le t]\nonumber \\&\quad \Leftrightarrow P[t+\tau \le L_1 +\sum _{i=1}^k\left[ \sum _{j=1}^i(L_j+R_j)\le t ,t+\tau \le \sum _{j=1}^i(L_i+R_i)+L_{i+1})\right] \nonumber \\&\quad = P[t+\tau \le L_1]+\sum _{i=1}^k\left[ P\left( \sum _{j=1}^i\left( L_j+R_j\right) \le t, t+\tau \le \sum _{j=1}^i(L_i+R_i)+L_{i+1}\right) \right] \end{aligned}$$

The following condition is considered to derive the above expression in (7).

We assume that

$$\begin{aligned} P(L_i\ge \tau )\le 1. \end{aligned}$$

Then the above equation has the required solution to (8) according to the assumption given above,

8 $$\begin{aligned} =\overline{F_1}(t+\tau )+\sum _{i=1}^k\left[ K_i(t)-(K_i*F_{i+1}(\cdot +\tau )(t)\right] . \end{aligned}$$

Therefore from Eqs. (8) and (9) we get

9 $$\begin{aligned} P[X(\nu )=1,t\le \nu \le t+\tau , \zeta > t]=\overline{F_1}(t+\tau )+\sum _{i=1}^k\left[ K_i(t)-(K_i*F_{i+1}(t+\tau )(t)\right] . \end{aligned}$$

Hence from the Eqs. (5), (6) and (9), we complete our proof.

### **Corollary 1**

*Assume the following conditions are satisfied:*

- (i) *Give a continuous functionsf*(*t*) *andg*(*t*) *defined on interval* (0, *T*] *where zero will not be taken into consideration.*
- (ii) *If*$T> 0$*for*$T\equiv \inf \{t>0 : f(t)=m(t)\}$, *then*$f(t)>m(t), \; \forall t \in (0,T]$.
- (iii) *If*$\tau >0$, *then*$S_\tau (t)>f(t)$*given that*$\forall \omega \in (0,t-\zeta ]$.

*where *$t-\zeta$* is always nominated as new repairable time of the system availability.*

### **Corollary 2**

*Assume that*$F \ge G$*defined in failure rate order. Then we get the following inequality,*

$$\begin{aligned} S_F(\tau ,\delta )\ge S_G(\tau ,\delta ),\quad \forall \tau \ge 0,\delta \ge 0. \end{aligned}$$

*where*$S_F(\tau ,\delta )$*and*$S_G(\tau ,\delta )$*are defined as the stable interval of system availabilities with lifetime distributionsFandGrespectively.*

### *Proof*

By using the properties of $F\ge G$ defined in failure rate order, this implies that

$$\begin{aligned} F_e \ge G_e \end{aligned}$$

in stochastically order or simply in stochastically greater than, where $F_e$ and $G_e$ are known as the equilibrium distribution function defined on *F* and *G* respectively. Unfortunately the case of stable point availability $F\ge G$ in stochastically order is not confirmed. Therefore, in general and necessarily, this implies that

$$\begin{aligned} S_F(\tau ,\delta )\ge S_G(\tau ,\delta ), \quad \forall \tau \ge 0, \delta \ge 0. \end{aligned}$$

Hence, the Corollary is proved.

### **Theorem 2**

*LetF*(*t*) *andG*(*t*) *satisfies the conditions given in corollary* 1 *in such that*$\forall \ t\ \in [0,T]$, *then*$m(t)=(f*g)(t)\le f(t)$. *Hence*$S_\tau (t)\ge m(t)\ge f(t)$.

*Therefore*$S_\tau (t)$*is decreasing from the interval*$t \in [0,T]$.

### *Proof*

We let $S_\tau (t)$ being the unique solution to the Eq. (1) above, $S_\tau (t)$ is derived as the following

$$\begin{aligned} S_\tau (t)=S_\tau ^{'}(t) \end{aligned}$$

where $S_\tau (t)$ is taken as a solution to

$$\begin{aligned} S_\tau (t)=[m(t)-f(t)]+\int _0^t S_\tau (t-\varsigma )h(\varsigma ){\mathrm {d}}\varsigma . \end{aligned}$$

Therefore

10 $$\begin{aligned} S_\tau (t)=[m(t-\zeta )-f(t-\zeta )]+\int _0^{t-\zeta }S_\tau (t-\zeta -\varsigma )h(\varsigma ){\mathrm {d}}\varsigma . \end{aligned}$$

Note from our assumption

$$\begin{aligned} \delta (t)=m(t-\zeta )-f(t-\zeta )\le 1, \quad \forall \ t\in [0,T], \end{aligned}$$

we have

$$\begin{aligned} N(t)=\sum _{t=0}^\infty K^{k+1}(t), \quad where \ \ k+1=n \end{aligned}$$

as $t=n$, having convolution functions as *K*(*t*) for $\zeta \le t$.

Then it is followed that

$$\begin{aligned} \delta (t)\le M(t)=(F*G)(t). \end{aligned}$$

Therefore, the following inequality is obtained

$$\begin{aligned} m(t)\le S_\tau (t). \end{aligned}$$

We conclude that, Eq. (10) above is defined by the repairable system which shows that $S_\tau (t)$ is decreasing function of

$$\begin{aligned} M(t)=(F*G)(t), \quad \forall \ t\in [0,T]. \end{aligned}$$

Hence, theorem is proved.

## Bounded for availability system

In this section, we provide some instant availability of $S_\tau (t)$ with respect to the upper bound $\overline{F}(t)$. This can be applied to the Eq. (1) according to the upper bound definition of reliability system for instant system availability $S_\tau (t).$

### **Theorem 3**

*Letf*(*t*) *andg*(*t*) *be probability density function having (C.D.F) defined byF*(*t*), *G*(*t*) *respectively . If*$(T_i>0)$*where*$i=0,1,2,\ldots,n$.

*Then*$m(t)>f(t), \,\forall t\in [0,T]$. For *any *$t \in [0,T]$, *we obtain*

11 $$\begin{aligned} S_\tau (t)\le \frac{\overline{F}(t)}{\overline{M}(t)}. \end{aligned}$$

### *Proof*

Given that $t \in [0,T]$ as from the Eq. (1), we get the relation

$$\begin{aligned} S_\tau (t)=\overline{F}(t)+\int _0^{t-\zeta }S_\tau (t-\varsigma ) {\mathrm {d}}\overline{M}(\varsigma )\le \overline{F}(t)+\int _0^{t-\zeta } S_\tau (t){\mathrm {d}}\overline{M}(\varsigma ) =\overline{F}(t)+S_\tau (t)M(t). \end{aligned}$$

For $\varsigma \le t$ and from the Corollary 1, it follows from Theorem 2 results that, the inequality above satisfies an upper bound on the $S_\tau (t)$.

Therefore the bound with $S_\tau (t)$ lies on the interval $[0,T_i]$ where $[i=0,1,\cdots n]$, $\forall T_i>0$.

Hence, the Theorem is proved.

### **Theorem 4**

*Suppose that*$T_i\ge 0.$*From the relation* (11), *the following inequality is satisfied.*

$$\begin{aligned} S_\tau (t)\ge \max _{0\le x\le T_i}\frac{\overline{F}(x)}{\overline{M}(x)}, \quad \forall 0\le t\le T_i \end{aligned}$$

### *Proof*

$S_\tau (t)$ is a solution to the given equation defined in (1).

Let us consider

$$\begin{aligned} Z(t)=C=\max _{0\le x \le T_i}\frac{\overline{F}(x)}{\overline{M}(x)}. \end{aligned}$$

This result comes from

$$\begin{aligned} Z(t)=C\overline{M}(t)+\int _0^{t-\zeta }Z(t-\varsigma ) {\mathrm {d}}M(\varsigma ), \quad \forall P[L_i\le Z(t)]\le 1. \end{aligned}$$

We present $S_\tau (t)-C=\delta (t)$ as unique solution to

$$\begin{aligned} \delta (t)=[\overline{F}(t)-C\overline{M}(t)]+\int _0^{t-\zeta } \delta (t-\varsigma ){\mathrm {d}}M(\varsigma ), \quad \forall t-\zeta \ge 0. \end{aligned}$$

Give that $$\overline{F}(t)-C\overline{M}(t)\ge 0, \forall \ 0\le t\le T_i$$.

Hence, $\delta (t)\ge 0,$ and the conclusion is that, the instant system availability $S_\tau (t)$ decrease over time *t*.

### *Remark*

We note that, the method used in the analysis of instant system availability, can be useful in the study of the measure-valued Markov process involved in the stochastic logistic model. which can be seen in Shang (2013)

## Comparison of availability system

Here we develop the availability of two state systems nominated as *E* and $E^\star$ as the lifetime as well as the repair time of $E^\star$ are denoted as the contradict of *E*. Different articles have provided this comparison for example in Cha et al. (2004) the measures are compared based on rate orderings has been failed , repairs, repair policy ordering and classifications of life distribution. It is also studied in Mi (1998), Biswas and Sarkar (2000) where the system availability is compared with respect to the perfect repair policy. In our case, the comparison of two systems is made by using the proof of the Theorems below in (5) and (6) with consideration of repair time that have been proposed in Theorem 1 of this article.

### **Theorem 5**

*Given that*$L+R=L^\star +R^\star$*as*$L\le L^\star$, *this is*$S_\tau (t)\le S_\tau ^\star (t), \; \forall t \ge 0.$

### *Proof*

From a small change says $\tau$ where $L+R=L^\star +R^\star$, we obtain

$$M(t)=M^\star (t), \quad \forall t\ge 0.$$

It implies that,

12 $$\begin{aligned} S_\tau (t)=\overline{F}(t)+\int _0^{t-\zeta } \overline{F}(t-\varsigma ){\mathrm {d}}N(\varsigma ) \end{aligned}$$

for $N(\varsigma )=\sum _{j=1}^\infty M^{(j)}(\varsigma )$, where $M^{(j)}$ is defined as the *k*-fold convolution of *M*.

In the same way as the above equation in (12) we get

13 $$\begin{aligned} S_\tau ^\star (t)=\overline{F^\star }(t)+\int _0^{t-\zeta } \overline{F^\star }(t-\varsigma ){\mathrm {d}}N^\star (\varsigma ). \end{aligned}$$

Thus

$$\begin{aligned} N^\star (\varsigma )=\sum _{j=1}^\infty M^{\star (j)}(\varsigma )=\sum _{j=1}^\infty M^{(j)}(\varsigma )=N(\varsigma ). \end{aligned}$$

Then from the Eqs. (13) and (14), we get

$$\begin{aligned} S_\tau ^\star (t)=\overline{F^\star }(t)+\int _0^{t-\zeta } \overline{F^\star }(t-\varsigma ){\mathrm {d}}N^\star (\varsigma )\ge \overline{F}(t)+\int _0^{t-\zeta }\overline{F} (t-\varsigma ){\mathrm {d}}N(\varsigma )=S_\tau (t). \end{aligned}$$

Therefore $L\le L^\star ,$ which indicates that $\overline{F}(t)\le \overline{F^\star }(t)\quad \forall t \ge 0$ since $S_\tau ^{\star }\ge S_\tau (t).$

### **Theorem 6**

*Suppose that for some*$T\ge 0.$*We have*

$$\begin{aligned} \overline{F}(t)\le \overline{F^\star }(t),\quad \forall T\in [0,t], \end{aligned}$$

*such that the convolution system*

$$\begin{aligned} m(t)\le m^\star (t). \end{aligned}$$

*And for any constant bwe have*

$$\begin{aligned} |m(t)|\le b \in [0,t_0]. \end{aligned}$$

*Therefore*

$$\begin{aligned} \int _0^{t-\zeta }|\overline{F}(t)|{\mathrm {d}}t\le \infty . \end{aligned}$$

*Hence*

$$\begin{aligned} S_\tau (t)\le S_\tau ^\star (t), \quad \forall t\in [0,T]. \end{aligned}$$

### *Proof*

To prove this Theorem, we have referred to the different theory related to the existence and uniqueness solution of an integral equation founded in Bellman and Cooke (1963) as defined in above relation (1). That is,

$$\begin{aligned} S_\tau (t)=\overline{F}(t)+ \int _0^{t-\zeta }m(t)\overline{F}(t-\varsigma ){\mathrm {d}}N(\varsigma ), \quad \forall \ \ 0\le t\le T. \end{aligned}$$

Therefore

$$\begin{aligned} |m(t)| &\le b+\int _0^{t-\zeta }|m(\varsigma )|| \overline{F}(t-\varsigma )|{\mathrm {d}}\varsigma \\& \le b + m(t)\int _0^{t-\zeta }\left| m(\varsigma )\right| {\mathrm {d}}\varsigma . \end{aligned}$$

It is given that, the below exponential expression is taken into account,

$$\begin{aligned} \int _0^{t-\zeta }|\overline{F}(\varsigma )|{\mathrm {d}}\varsigma \le b \exp \left[ \int _0^{t-\zeta }h(\varsigma ){\mathrm {d}}\varsigma \right] \int _0^{t-\zeta }\exp \left[ -\int _0^{t-\zeta }m(\varsigma ) {\mathrm {d}}\varsigma \right] {\mathrm {d}}\varsigma . \end{aligned}$$

If *m*(*t*) is bounded, then the solution exist and we get $m(t)=m^\star (t)$.

For a small change of time $\tau \in [0,t]$, we get

$$\begin{aligned} S_0(t)=\overline{F}(t),\ldots , S_i(t)=\overline{F}(t)+\int _0^{t-\zeta }m(\varsigma )S_{i-1} (t-\varsigma ){\mathrm {d}}\varsigma \end{aligned}$$

for all $$1\le i\le n+1.$$

We use the successive approximation to the instant system availability and then we define

$$\begin{aligned} \quad S_0(t)&=m(t),\\ S_1(t)&=m(t)+\int _0^{t-\zeta }m(\varsigma )S_0(t-\varsigma ){\mathrm {d}}\varsigma ,\\ S_2(t)&=m(t)+\int _0^{t-\zeta }m(\varsigma )S_1(t-\varsigma ){\mathrm {d}}\varsigma ,\\ & \qquad\qquad\quad \vdots \\ S_{n+1}(t)&=m(t)+\int _0^{t-\zeta }S_n(t-\varsigma )\zeta (\varsigma ){\mathrm {d}}\varsigma . \end{aligned}$$

Proceeding in similar manner, we can write the expression as follows

$$\begin{aligned} S_{n+1}(t)-S_n(t)=\int _0^{t-\zeta }\left[ S_n(\varsigma ) -S_{n-1}(\varsigma )\right] m(t-\varsigma ){\mathrm {d}}\varsigma . \end{aligned}$$

Then by using the inductive method, we get the following relations

14 $$\begin{aligned} |S_1(t)-S_0(t)| &\le bt_0 m^\star (t)\\ |S_2(t)-S_1(t)| &\le bt_0 m^\star (t)\int _0^{t-\zeta }h^\star (s){\mathrm {d}}\varsigma \\ |S_2(t)-S_1(t)| &\le bt_0 m^\star (t)\int _0^{t-\zeta }m^\star (\varsigma )\left[ \int _0^{t-\zeta }m^\star (s_1){\mathrm {d}}s_1\right] {\mathrm {d}}\varsigma \\ &\le bt_0\frac{m^\star (t)}{2!}\left[ \int _0^{t-\zeta }m^\star (\varsigma ){\mathrm {d}}\varsigma \right] ^2\\&\quad\qquad\quad \vdots \\ |S_{n+1}(t)-S_n(t)|&\le bt_0\frac{m^\star (t)}{n!}\left[ \int _0^{t-\zeta }m^\star (\varsigma ){\mathrm {d}}\varsigma \right] ^n. \end{aligned}$$

Therefore, from the above expression (14), we get the following

$$\begin{aligned} S_\tau (t)=\lim _{n\rightarrow \infty }S_n(t) \ \ {\rm and} \ \ S_\tau ^\star (t)=\lim _{n\rightarrow \infty }S_n^\star (t), \ \ \ \forall t \in [0,T]. \end{aligned}$$

Then by induction method we have

$$\begin{aligned} S_n(t)\le S_n^\star (t), \quad \forall t\in [0,T] \end{aligned}$$

Therefore, we get

15 $$\begin{aligned} S_\tau (t)=\lim _{n\rightarrow \infty }S_n(t)\le \lim _{n\rightarrow \infty } S_n^\star (t)=S_\tau ^\star (t). \end{aligned}$$

Which implies that

$$\begin{aligned} S_\tau (t)=S_\tau ^\star (t). \end{aligned}$$

Hence, the Theorem is proved.

## Numerical examples for clarification

In this section, we use some examples to find a solution to the Eq. (1) by testing three distinct types of the distribution function, such as Gamma, Log-normal and Weibull distributions, where we make a combination of two between them. Related studies have been done in Mishra and Jain (2013) where they have obtained the four individual cases of the repair distribution namely exponential, Gamma, Weibull and Pareto have been examined numerically for the illustration purposes, we have been referred by so many authors talked about illustration of numerical examples specifically in Huang and Mi (2013, 2015) where the various examples taken, have shown the high accuracy and efficiency. From those experiences, we can determine the value of $t=T$ defined in Theorem 2 and each figure associated with these examples. All these figures clearly indicate that the interval determined by Theorem 2, shows that $S_\tau (t)$ decreases and it is also very sharp.

### *Example 1*

Suppose that $L\sim \ln N(\mu ,\sigma )$ and $R\sim Weibull(a,\lambda )$ be defined by density functions respectively given by

$$\begin{aligned} f(t)=\frac{1}{t\sigma \sqrt{2\pi }}e^-{\frac{(\ln t-\mu )^2}{2\sigma ^2}}\ \ \quad {\rm and}\quad \ \ g(t)=\frac{a}{\lambda }\left( \frac{x}{\lambda }\right) ^{a-1} e^-\left( \frac{x}{\lambda }\right) ^a. \end{aligned}$$

**Fig. 1:**
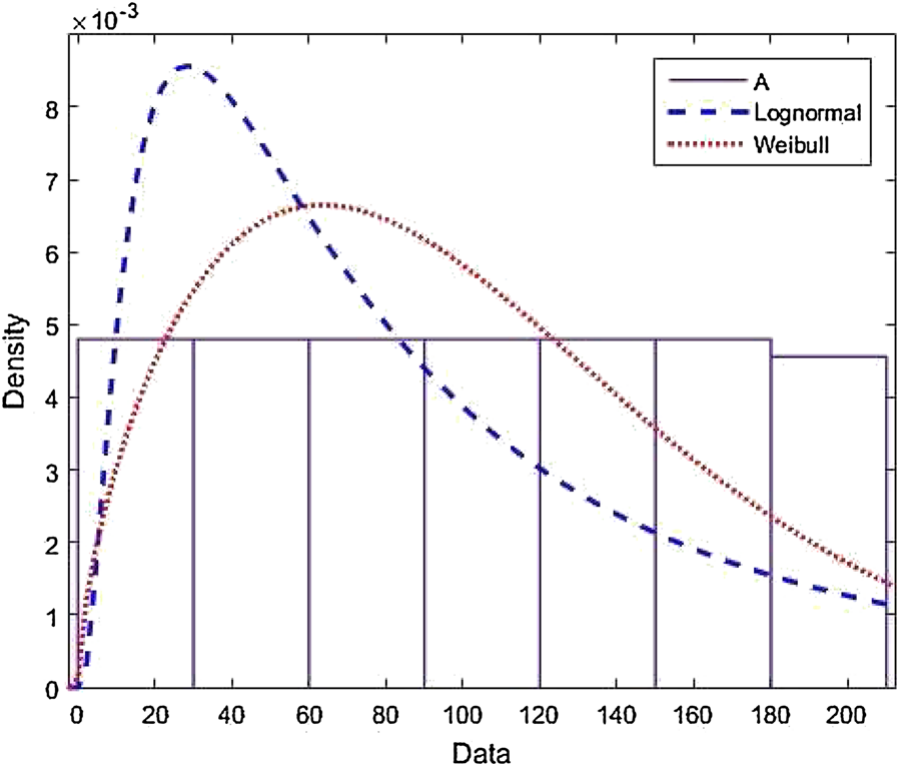
$Example 1: L\sim Lognormal, R\sim Weibull.$ This Figure show that $S_\tau (t)$ decrease in the given interval

We choose $\mu =4$, $\tau =0.25$ and $a=115$, $\lambda =1.62$ in this example. In Fig. 1 below, the curve of $S_\tau (t)$ is obtained from the numerical solution is shown. The given example demonstrates that the result of Theorem 2 is very sharp fall. In fact, in this example the value of *T* is between 58.60 and 58.61, while the value of $T_0$ from the same Theorem are between 58.56 and 58.60 which is just slightly smaller than *T*, this also shows that $S_\tau (t)$ decreases in the given interval above.

### *Example 2*

**Fig. 2:**
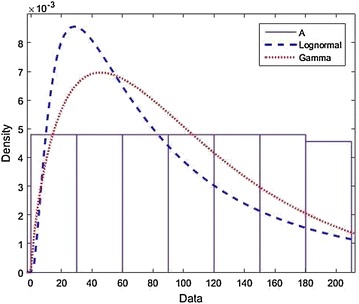
$Example 2: L\sim Lognormal, R\sim Gamma.$ This Figure compare $S_\tau (t)$ with the lower bound $\frac{\overline{F}(t)}{\overline{H}(t)}$ given in Theorems 3 and 4

Let $L\sim \ln N(p,\alpha )$ and $R\sim Gamma(\mu ,\sigma ),$ by choosing $p=1.7$, $\alpha =58$, $\mu =4$ and $\sigma =0.25$, we compare $S_\tau (t)$ with the lower bound $\frac{\overline{F}(t)}{\overline{H}(t)}$ given in Theorems 3 and 4. The functions $S_\tau (t)$ and $\frac{\overline{F}(t)}{\overline{H}(t)}$ is plotted in Fig. 2 below. When $t<T=58.6$, they are looking almost same.

### *Example 3*

Given that the lifetime *L* and repair time *R* have cumulative distribution functions *F* and *G* respectively. We extend the availability of the system as $S_F(t)$ in order to make an accent it dependance on distribution functions *F*. It is well interested to state that $S_F(t)$ would be expended on high value if the lifetime $L\sim Gamma(p,\alpha )$ and $R\sim Weibull(a,\lambda )$ with having density function respectively given by

$$\begin{aligned} f(t)=\frac{\alpha ^p}{\sqrt{p}}e^{-\alpha t}t^{p-1}\ \quad {\rm and}\quad \ g(t)=\frac{a}{\lambda }\left( \frac{x}{\lambda }\right) ^{a-1} e^-\left( \frac{x}{\lambda }\right) ^a. \end{aligned}$$

**Fig. 3:**
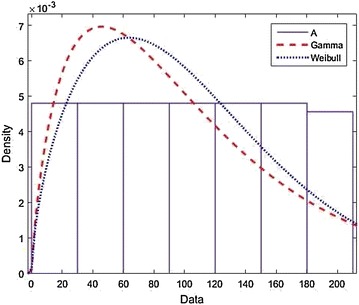
$Example 3: L\sim Gamma, R\sim Weibull.$ This Figure emphasizes that the instant system availability $S_\tau (t)$ is well decreasing

From the Fig. 3 below, it is observed that the two curves of system availabilities present the corresponding value which is $p=1.7$ and $p=3.4$ cross each other at least two times even if $Gamma(1.7,\alpha )\le Gamma(3.4,\alpha )$, this emphasizes that the instant system availability $S_\tau (t)$ is well decreasing.

## Application to bathtub

From the different literature review, many different schemes and practical examples, the estimation was carried out by assuming that, the sequence of failure and repair time are two independent sequences of *i*.*i*.*d* random variables. The details applications of this article have been found in Rausand and Høyland (2004), Barlow and Hunter (1960) Wong et al. (1999).

However, the given assumption need not hold in many situations while the repair times may depend on the previous failure time due to the influence of the operation environment of the system is applied.

This simple article has found the applications for a variety of some fields such as Reliability Engineering, Industrial economic systems, Maintenance management system, mechanical engineering. And it is may specially useful application in Measure-valued Markov process involved in the stochastic logistic model in Shang (2013) as noticed above in bounded availability system section.

In this part of application, we assume that the lifetime distribution of the instant system availability has bathtub-shaped failure rate function of $t_1\le t_2$ as its change points, this has been referred in Mi (1995b, 1998).

Frequently we can apply a burn-in procedure to improve the availability of the instant system, and it has been observed that the burn-in times that optimise different availability or reliability characteristics never go beyond to the first change point $t_1$.

For more details of bathtub-shaped failure rate function and optimal burn-in times are discussed here (Lai and Xie 2006; Block et al. 1994). From the definition of bathtub-shaped failure rate distribution function said that, a failure rate distribution function *r*(*t*) is said to have a bathtub-shaped with change point $t_1\le t_2$ if *r*(*t*) is strictly decreases in $t\in (0,t)$, is a constant on $(t_1,t_2)$ and strictly increasing to $t\in (t_1,\infty ).$ Using this definition together with the combination of the above previous comparison and examples results sections respectively, the following Theorem 7 below is a useful application of this article.

### **Theorem 7**

*Given that the lifetime distribution functionFof a system has a bathtub-shaped failure rate functionr*(*t*) *with its change point*$t_1\le t_2.$*Assume that*$F_b$, $\overline{F}_b$*where*$\overline{F}= 1-F$*is the distribution and survival function of the instant system surviving burn-in timeb*. *This means that*

$$\begin{aligned} \overline{F}_b(t)=\frac{\overline{F}(b+t)}{\overline{F}(b)}, \quad \forall t \ge 0. \end{aligned}$$

*Therefore for each of the following maximization problems, the optimal burn-in can not exceed*$t_1$*such as:*

(i) $$max_{b\ge 0} S_b(\tau ,\delta ) = max_{b\ge 0}(\frac{\mu (b)}{(\mu (b)+\delta )}),$$*where*$$\mu (b)=\int _0^{\infty }\overline{F}_b(t){\mathrm {d}}t$$
(ii) $max_{b\ge 0} S_b(\tau ,\delta ) = max_{b\ge 0}(\frac{1}{(\mu (b)+\delta )})\int _\tau ^{\infty }\overline{F}_b(t){\mathrm {d}}t$ for any $\tau \ge 0$ and $\delta \ge 0.$
(iii) $$max_{b\ge 0} \overline{S}_b = max_{b\ge 0} ((\int _0^{\infty }\overline{F}_{bp}(t){\mathrm {d}}t + \mu _X \int _0^{\infty } q(t)\overline{F}_{bp}(t){\mathrm {d}}t +\mu _Y))$$* where*$$\overline{F}_{bp}$$

*is defined as the following situation*

$$\begin{aligned} F_{bp}(t) &= exp\left\{ -\int _0^t p(u) r_b(u){\mathrm {d}}u\right\} ,\\ r_b(t) &= r(b+t), \end{aligned}$$

*this is known as the failure rate function of*$F_b(t)$* and where*$0\le p(t)<1$* is assumed as an increasing function satisfying*

$$\begin{aligned} \int _0^{\infty } p(t) r(t){\mathrm {d}}t = \infty . \end{aligned}$$

### *Proof*

The proof of (*i*). Let us express the burn-in time which maximizes the mean residual lifetime $\mu _b$ by $b^\star .$ Therefore it is positively observed that,

$$\begin{aligned} b^\star \le t_1 \end{aligned}$$

is required. From this and the fact that the following given expression of

$$\begin{aligned} \frac{\varsigma }{(\varsigma +\delta )} \end{aligned}$$

is known as a strict increasing function of $\varsigma ,$ this immediately implies the given proof of (i).

The proof of (ii) is obtained as the following. By the assumptions and properties that the failure rate *r*(*t*) is a bathtub-shaped and having $t_1$ as its first change point as defined above, it implies the following inequality defined as

$$\begin{aligned} r_{t_1}(t)\le r_b(t), \quad \forall b\ge t_1,\; \forall t\ge 0. \end{aligned}$$

Refer to the given above Corollary 2, we conclude that

$$\begin{aligned} F_{t_1}\ge F_b, \quad \forall b\ge t_1 \end{aligned}$$

is satisfied.

Hence

$$\begin{aligned} S_{F_{t_1}}(\tau ,\delta )> S_{F_b}(\tau ,\delta ),\quad \forall \tau>0,\quad \forall \delta \ge 0,\; b>t_1 \end{aligned}$$

is well verified.

Therefore, for any given parameters $\tau >0$ and $\delta >0$, the function of the burn-in times *b*, $S_b(\tau ,\delta )$ is always tested as continuous on the given interval of $[0,t_1].$

Also, the burn-in times $b^{\star }$ satisfies

$$\begin{aligned} S_{b^{\star }}(\tau ,\delta ) =max_{0\le b \le t_1}S_b(\tau ,\delta )= max_{b\ge 0}S_b(\tau ,\delta ). \end{aligned}$$

Which is entirely less than $t_1.$ Hence, the relation (ii) is proved.

Refer to the above proof of the relation (ii) given in Theorem 7, the proof of (iii) in the same Theorem 7 is obtained by using the same method. Therefore the relation (iii) is true.

Hence, the Theorem is proved.

Briefly, many statistical ageing concepts have been applied to describe the instant system availability component ages with time, it have observed that with the probability sense, the lifetime of the scheme component tends to decrease accordingly. By the above example of the application to the bathtub, this have also been covered by the monotonicity condition as well as numerical examples section respectively discussed above in this article has shown that the instant system availability $S_\tau (t)$ decrease.

Consequently, the given results provided above by this article are adjusted and justified.

## Conclusion

Instant System availability denoted as $S_\tau (t)$ of a repairable system is considered as the relevant estimate measure of it system performance. It is the case that $S_\tau (t)$ does not have closed form of expression of the literature attention focuses on the steady-state availability $S_\tau (\infty )$. Different from the most literature, this article provide analysis of various properties of the instant system availability $S_\tau (t)$ as a function of time instead of the steady state availability. An interval where $S_\tau (t)$ monotonically decreases is obtained, upper bound to $S_\tau (t)$ is derived, both of them have been well clarified in the numerical examples and some comparison results regarding availabilities of different systems are proved. Several examples which validate the analytically derived properties of $S_\tau (t)$ are also shown above. The details applications of this article are also strongly discussed in "Application to bathtub" section. We have studied the instant system availability by using the univariate (*i*.*i*.*d*) sequence, from this if the failure and repair times form a bivariate (*i*.*i*.*d*) sequence, the estimation of availability measures, point availability, average availability and interval reliability is interesting research issue which can be taken to be addressed. Further more, the inspection is very essential for keeping away a system components from damage. Hence different conditions based on maintenance will be taken as a suitable to investigate in future research. Certainly some given possible generalization could be explored for instance many questions regarding to the mechanism of choosing a complete or a minimal repair are still open and further research can be considered as principal.
